# Supplementary figures and images for: Identification of novel genetic susceptibility loci for thoracic and abdominal aortic aneurysms via genome-wide association study using the UK Biobank Cohort
Source: PLoS One. 2021 Sep 1;16(9):e0247287. doi: 10.1371/journal.pone.0247287 (PMC8409653; doi:10.1371/journal.pone.0247287)

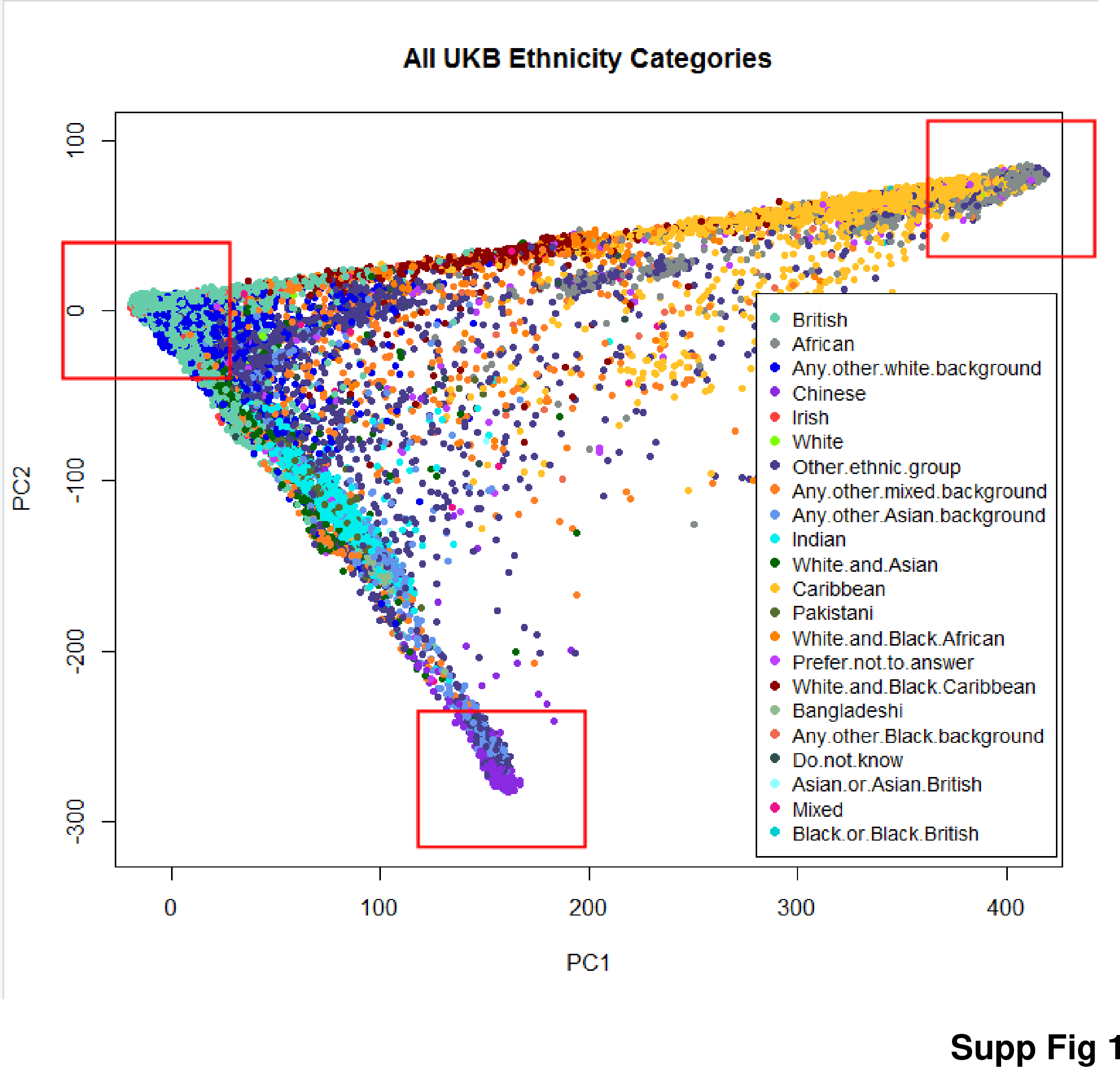

Supplement: S1 Fig — When selecting controls for comparison with cases, control subjects were picked from subjects within 80 units on the PC1 vs. PC2 graph. The size of 80 units is illustrated with the red boxes around subjects who are primarily European, Chinese or African Ethnicity based on the PC1 and PC2 eigenvalues provided by the UK Biobank. (TIF) [file pone.0247287.s001.tif]

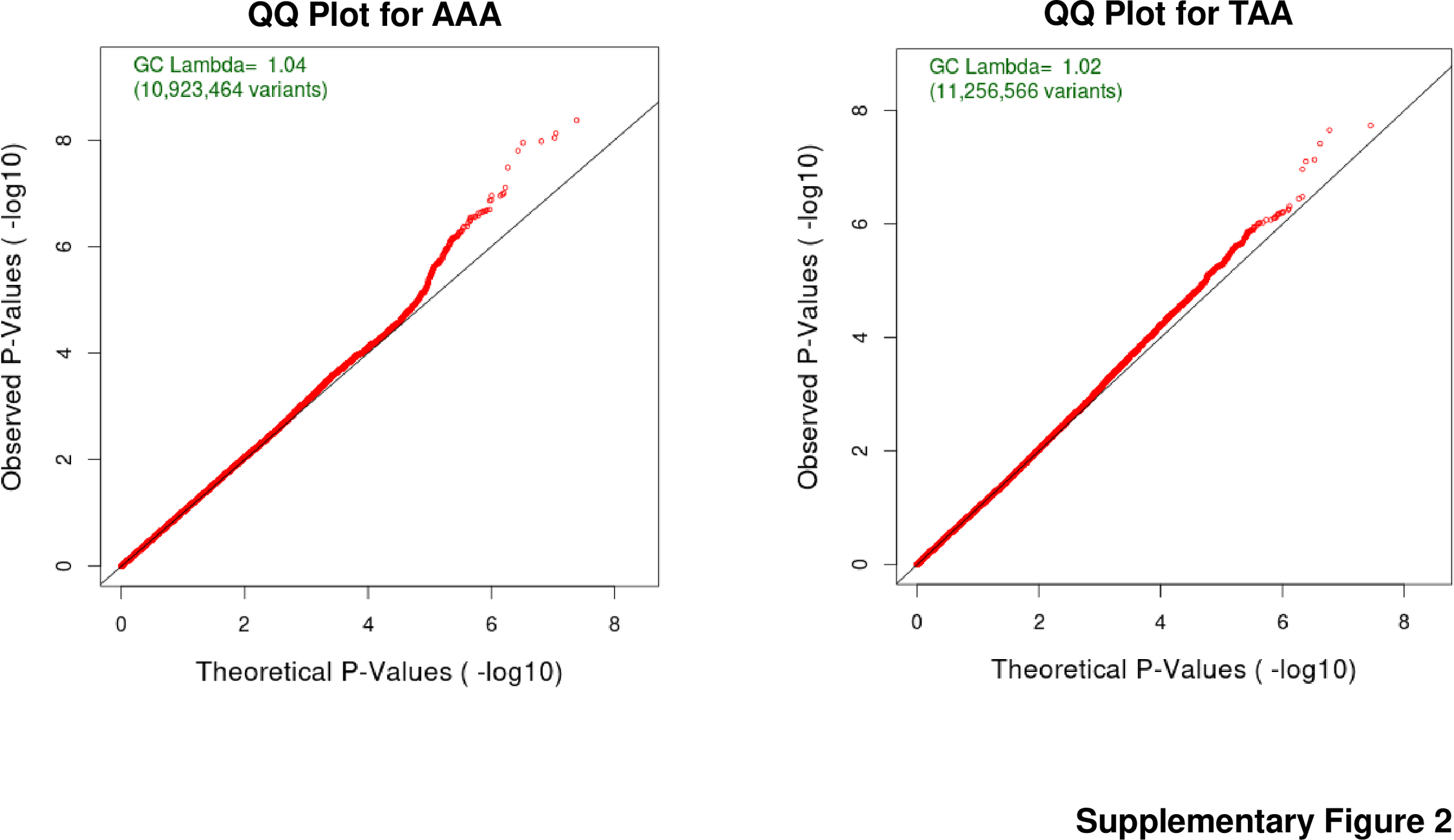

Supplement: S2 Fig — The genomic control (GC Lambda) values of 1.04 (AAA) and 1.05 (TAA) are within the generally accepted range for GWAS. (TIF) [file pone.0247287.s002.tif]

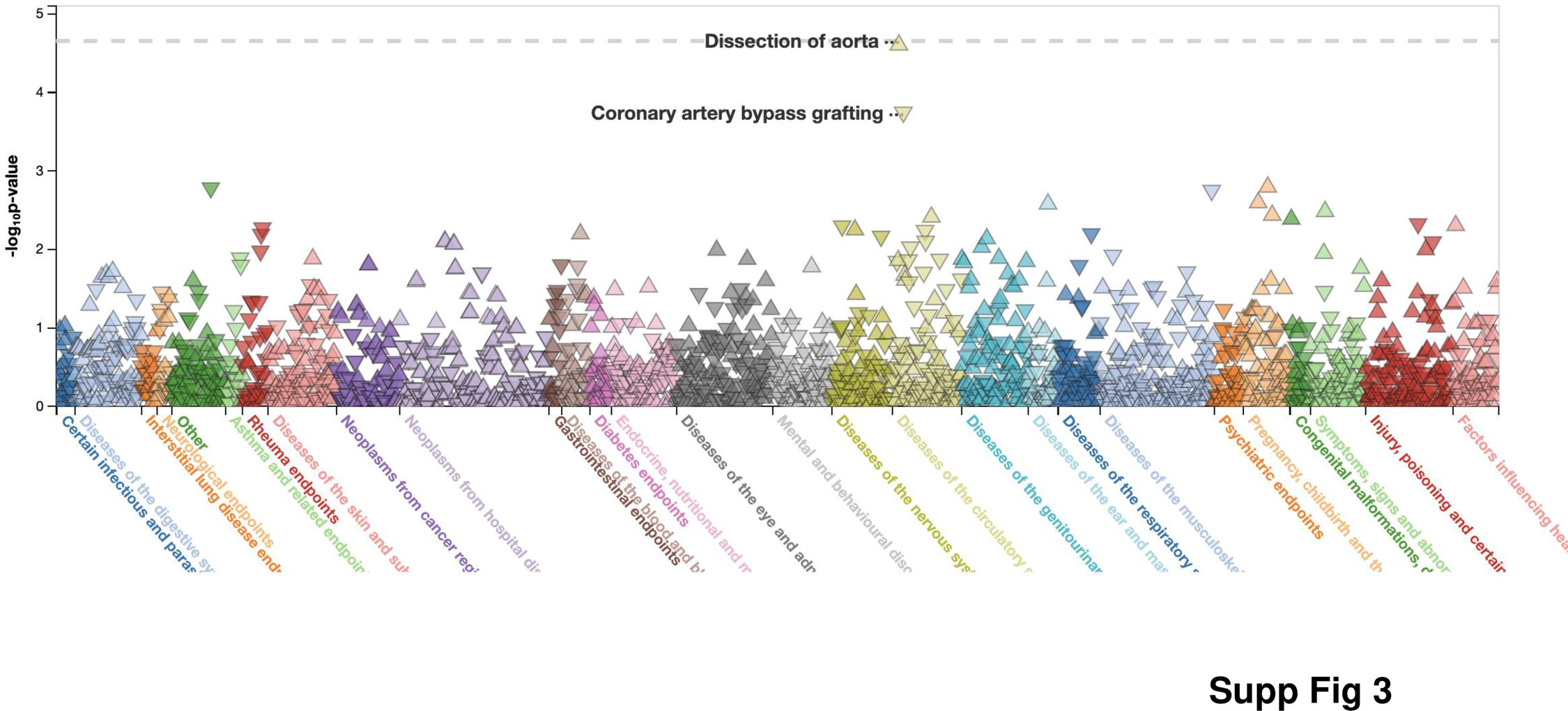

Supplement: S3 Fig — Manhattan plot of phenome wide association study (PheWAS) is shown. (TIF) [file pone.0247287.s003.tif]

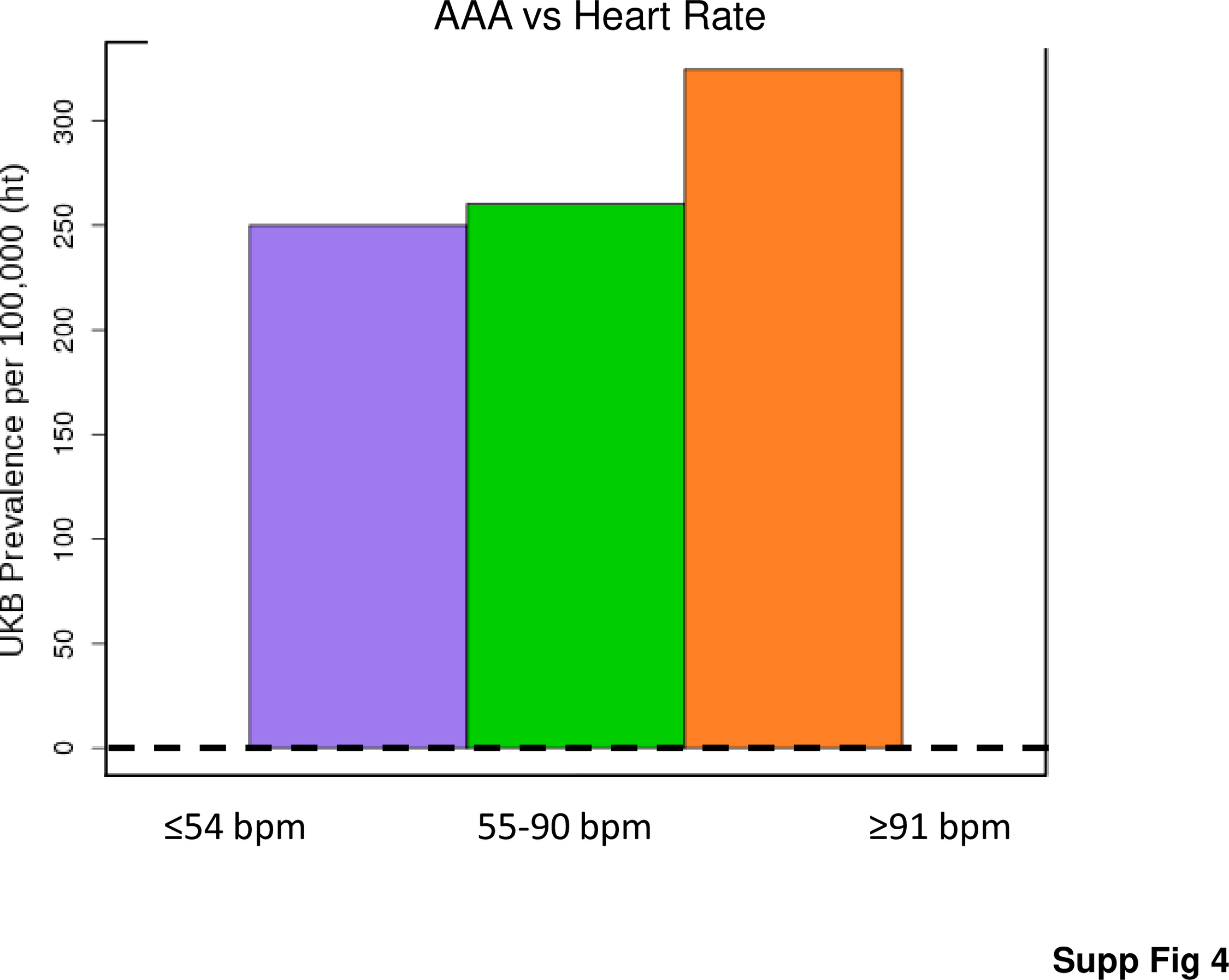

Supplement: S4 Fig — A general trend toward slightly increased AAA prevalence is seen with tachycardia. (TIF) [file pone.0247287.s004.tif]
